# Role of mitochondria in apoptotic and necroptotic cell death in the developing brain

**DOI:** 10.1016/j.cca.2015.01.026

**Published:** 2015-12-07

**Authors:** Claire Thornton, Henrik Hagberg

**Affiliations:** a Centre for the Developing Brain, Division of Imaging Sciences and Biomedical Engineering, King's College London, King's Health Partners, St. Thomas' Hospital, London SE1 7EH, United Kingdom; b Perinatal Center, Department of Clinical Sciences & Physiology and Neuroscience, Sahlgrenska Academy, Gothenburg University, Sweden

**Keywords:** AMPA, α-amino-3-hydroxy-5-methyl-4-isoxazolepropionic acid, Apaf-1, apoptotic protease activating factor 1, CAD, caspase-activation DNase, cyt c, cytochrome c, Cy, cyclophilin, Drp-1, dynamin-related protein 1, ENDO, endonuclease, AIF, apoptosis-inducing factor, ATP, adenosine triphosphate, MOMP, mitochondrial outer membrane permeabilization, MP, mitochondrial permeabilization, NMDA, N-methyl-D-aspartate, HI, hypoxia–ischemia, LPS, lipopolysaccharide, MLKL, mixed lineage kinase domain-like protein, NO, nitric oxide, RIP, receptor-interacting serine/threonine-protein kinase 1, ROS, reactive oxygen species, TNF, tumor necrosis factor, TLR, Toll-like receptor, TRADD, tumor necrosis factor receptor type 1-associated DEATH domain, TRIF, TIR-domain-containing adapter-inducing interferon-β, TRAIL, TNF-related apoptosis-inducing ligand, TWEAK, tumor necrosis factor (ligand) superfamily, member 12, Perinatal brain injury, Hypoxia–ischemia, Mitochondria, Apoptosis, Necroptosis, Necrosis

## Abstract

Hypoxic–ischemic encephalopathy induces secondary brain injury characterized by delayed energy failure. Currently, therapeutic hypothermia is the sole treatment available after severe intrapartum asphyxia in babies and acts to attenuate secondary loss of high energy phosphates improving both short- and long-term outcome. In order to develop the next generation of neuroprotective therapies, we urgently need to understand the underlying molecular mechanisms leading to cell death. Hypoxia–ischemia creates a toxic intracellular environment including accumulation of reactive oxygen/nitrosative species and intracellular calcium after the insult, inducing mitochondrial impairment. More specifically mitochondrial respiration is suppressed and calcium signaling is dysregulated. At a certain threshold, Bax-dependent mitochondrial permeabilization will occur leading to activation of caspase-dependent and apoptosis-inducing factor-dependent apoptotic cell death. In addition, hypoxia–ischemia induces inflammation, which leads to the release of TNF-α, TRAIL, TWEAK, FasL and Toll-like receptor agonists that will activate death receptors on neurons and oligodendroglia. Death receptors trigger apoptotic death via caspase-8 and necroptotic cell death through formation of the necrosome (composed of RIP1, RIP3 and MLKL), both of which converge at the mitochondria.

## Highlights

• Hypoxic-ischemic encephalopathy induces secondary brain injury characterized by delayed energy failure and excitotoxicity.
• Hypoxia-ischemia triggers accumulation of reactive oxygen species andintracellular calcium, which induces mitochondrial dysfunction.
• Mitochondrial impairment can cause Bax-dependent mitochondrial permeabilization, which triggers release of pro-apoptotic proteins and cell death.
• During the recovery phase, Inflammation is produced leading to death receptor activation and induction of necroptosis.

## 1 Introduction

The causes of neonatal brain damage in response to hypoxic–ischemic insult are multifactorial. In the developing brain, lack of oxygen availability results in an initial depletion of high energy phosphates, in particular ATP and phospho-creatine. These levels return transiently to baseline but are followed by a second more prolonged depletion of cellular energy reserves accompanied by progression of brain injury [1], [2]. These disturbances in energy metabolism trigger a number of pathophysiological responses but there is a common convergence at the level of the mitochondria. This range of injurious events includes the release of excitatory amino acids activating glutamate receptors (NMDA, AMPA), activation of nitric oxide synthase on neurons and oligodendroglial precursors, leading to increased intracellular Ca^2+^ and accumulation of reactive oxygen species (ROS) through release of nitric oxide (NO) [1], [3].

## 2 Effect of calcium on mitochondria

**Fig. 1:**
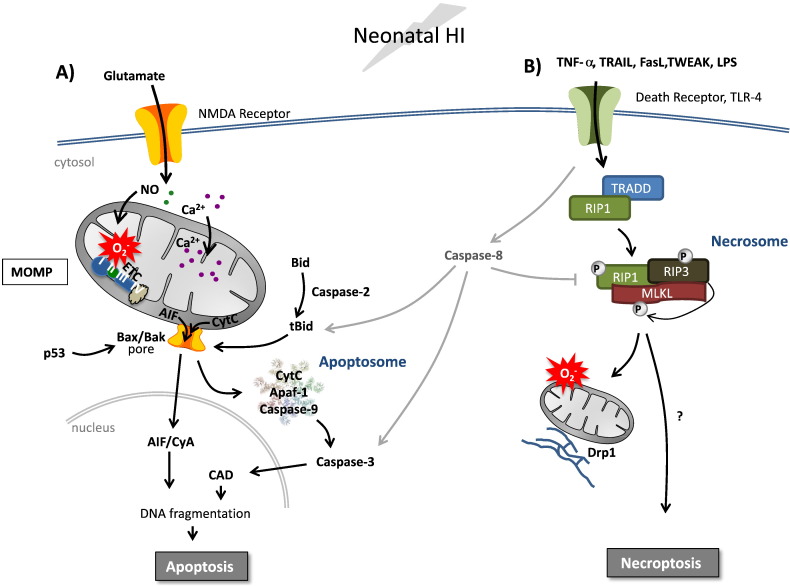
Interweaving apoptosis and necroptosis pathways after neonatal HI insult. A) Neonatal HI induces mitochondrial accumulation of calcium, increased production of reactive oxygen species, and suppression of mitochondrial respiration that culminates in MOMP. Changes in Bcl-2 family proteins induce Bax-dependent MOMP leading to the release of cytochrome c (cyt c) and apoptosis-inducing factor (AIF). Cyt c induces apoptosome formation leading to caspase-3 activation, caspase-activated DNase (CAD) and DNA degradation. AIF forms a complex with cyclophilin A (CyA) which translocates to the nucleus and induces chromatinolysis and apoptotic cell death. B) Concomitantly, inflammatory microglia and astroglia will release tumor necrosis factor-α (TNF-α) or other ligands (FasL, TWEAK, TRAIL and lipopolysaccharide, LPS) leading to the activation of death receptors, which in turn can induce both apoptosis and necroptosis depending on the availability of caspases. Recruitment of TRADD (or other adaptor proteins) and RIP1 will lead to caspase-8 activation and cleavage of Bid leading to apoptotic cell death. Alternatively, under conditions when caspase-8 is inhibited, TRADD facilitates the interaction and activation of RIP1 and RIP3. RIP3 phosphorylates and recruits MLKL to the necrosome which can then be targeted to both plasma and mitochondria-associated endoplasmic reticulum membranes triggering increased reactive oxygen species, fission and necroptosis. Alternative non-mitochondrial mechanisms may also play a role in the induction of necroptosis.

Activation of NMDA and AMPA receptors after HI (hypoxia–ischemia), in response to excitotoxic amino acid release, results in cellular uptake of calcium. Indeed, we have found increased deposits of intracellular calcium in the endoplasmic reticulum, cytosol, nucleus and more significantly in the mitochondrial matrix of neurons [4]. Not only does this influx activate a number of intracellular signaling pathways, it is taken up by mitochondria causing mitochondrial swelling, impairment of respiration, increased production of reactive oxygen species and may ultimately trigger mitochondrial permeabilization (MP) and cell death [1], [5], [6] (Fig. 1).

## 3 Mitochondrial permeabilization and apoptosis

Mitochondria determine cell fate in many different ways. They can induce cell death due to their ability to release proapoptotic proteins, which occurs following MP. MP can occur either through selective opening of the outer mitochondrial membrane, mitochondrial outer membrane permeabilization (MOMP), or be the result of opening of the mitochondrial permeability transition pore, which permeabilizes both the outer and inner mitochondrial membranes [7]. MOMP appears predominantly to induce apoptosis whereas mitochondrial permeability transition pore opening results in mitochondrial swelling and tends to lead to necrotic cell death. Importantly, cyclophilin D has been shown to be implicated in mitochondrial permeability transition pore opening in the adult brain after ischemia [8], whereas Bax-dependent MOMP seems to be the predominant mechanism in the neonatal brain following HI [9]. Mitochondria can also be involved in necroptosis (see below).

Mitochondrial permeabilization results in the release of key proapoptotic proteins cytochrome c, apoptosis inducing factor (AIF), endonuclease (endo) G and Smac/Diablo from the mitochondria to the cytosol [3], [5], [10], [11], [12]. Each protein has different downstream targets, but all contribute to cell death. Following translocation to the cytosol, cytochrome c binds to Apaf-1 forming an apoptosome which binds to procaspase-9 leading to caspase-3 activation [13]. Smac/Diablo also enhances the activity of caspases, whilst AIF, which is caspase-independent, interacts with cyclophilin A. This complex then translocates to the nucleus and is associated with DNA fragmentation which has been shown to occur following neonatal HI [10]. High expression of proapoptotic proteins such as caspase-3, Bax and Bcl-2 during development strongly suggests that apoptosis is more prominent in the immature brain compared with the adult [5], [11], [12].

### 3.1 Apoptosis and neonatal HI brain injury

The induction of MOMP in the immature brain after HI depends on Bcl-2 family proteins; Bax translocates from the cytosol to the mitochondria and in association with Bak forms pores in the outer membrane resulting in the subsequent release of proapoptotic proteins. In the immature brain, Bax and its associated proteins are highly expressed, with further upregulation of expression occurring following neonatal HI [9], [14], [15]. Pharmacological inhibition of Bax-dependent mitochondrial permeabilization prior to neonatal HI attenuates the severity of brain injury [16] highlighting that, in the immature brain, Bax-dependent MOMP is a critical event leading to execution of cell death.

One of the proposed regulators of Bcl-2 proteins includes the tumor suppressor p53, which has been shown to stimulate mitochondrial permeabilization and apoptosis thereby regulating cell death. Following activation, p53 accumulates in the nucleus and can upregulate proapoptotic genes such as Bax [17]. Consistent with the above observations that the development of perinatal brain injury is Bax- and MOMP-dependent, it has been shown that blocking mitochondrial p53 with the inhibitor pifithrin-μ, after neonatal HI in the rodent resulted in decreased lesion size and improved functional outcome [18]. However, pifithrin-μ also modulates other proteins (e.g. heat shock proteins) and genetic evidence is yet lacking that p53 is critical for triggering MOMP in the setting of perinatal brain injury.

The initiator caspase caspase-2, also triggers Bax-mediated MOMP [19], and data suggest that caspase-2 inhibition offers the potential for improved neuroprotection after perinatal HI. Newborn caspase-2 knockout mice were significantly protected in HI and excitotoxic models of neuronal damage [20], a protection which was additive when combined with hypothermia [21]. Furthermore, TRP601, a pharmacological caspase-2 inhibitor, reduced brain injury in three *in vivo* models of immature brain injury without adverse effects [22], [23].

## 4 Death receptors and necroptosis

Necrosis is defined as accidental uncontrolled cell death. On the other hand, necroptosis or programmed necrosis is a form of highly regulated cell death that morphologically resembles necrosis [24]. Necroptosis is activated in situations when the AIF- or caspase-dependent apoptotic pathway is inhibited by, for example, viruses or ATP deficiency. Necroptosis is commonly induced by death receptor ligands such as TNF-α, Fas, TRAIL or by Toll-like receptor (TLR) 3 and 4 signaling [25] (Fig. 1), and can occur following ischemia in adults [26] or hypoxia–ischemia in the immature brain [27]. Once ligand-death receptor binding occurs, an adaptor protein is recruited which varies depending on the receptor but can include TRADD (for TNF receptor) or TRIF (for TLR3/4). The adaptor promotes the interaction between two kinases, RIP1 and RIP3 (also known as RIPK1 and RIPK3) forming the key signaling complex, the necrosome. Recently, it was also discovered that the mixed lineage kinase domain-like protein (MLKL) is also critical in necroptosis as MLKL^−/−^ mice are unable to undergo necroptosis [28]. It is still not completely understood exactly how necrosome formation induces cell death. Phosphorylated RIP3 can recruit MLKL to the necrosome which promotes its translocation to mitochondrial-associated endoplasmic reticulum membranes [29] and that RIP3 induces a shift in metabolism leading to excessive ROS production and subsequent cell death [25] (Fig. 1). Another proposal is that assembly of the necrosome induces activation of Drp-1 (regulates mitochondrial fission), which somehow induces cell death as Drp-1 inhibitors block necroptosis [30]. These data strongly imply that the execution phase of necroptosis, similar to apoptosis, relies on mitochondria in some cell types, although mitochondria-independent mechanisms may also play a role [31].

Necroptosis and apoptosis are fundamentally linked as certain ligands can trigger both pathways. In this situation, caspase-8 activation state sits at the divergence point. RIP1 and RIP3 are substrates for cleavage by active caspase-8 and therefore necroptosis is inhibited [32]. Conversely, caspase-8 homodimers promote apoptosis whereas caspase-8-FLIP heterodimers inhibit necroptosis [33] (Fig. 1). In addition, RIP3 may also play a role in the decision of the cell to follow an apoptotic or necroptotic route although the mechanism is unclear [34], [35].

### 4.1 Necroptosis and HI injury

There is increasing evidence that death receptors are involved in immature brain injury [13], [36]. Children who develop cerebral palsy show increased blood levels of TNF-α [37], and TNF receptor 1 is critical for LPS-mediated sensitization to oxygen glucose deprivation *in vitro* [38]. Moreover, deletion of the TNF gene cluster abolishes LPS-mediated sensitization of the neonatal brain to HI insult [39]. The FasL binds with Fas death receptor triggering cell death [13]. HI activates Fas death receptor signaling in the neonatal brain and Fas receptor gene deficiency confers neuroprotection [40]. The death receptor agonists TRAIL and TWEAK have also been implicated in adult stroke models [41], [42] and we recently found that TRAIL–Death Receptor signaling is involved in hypoxic–ischemic brain injury [36].

Caspase-8 inhibition reduces HI brain injury in the neonate in some studies [43] suggesting that death receptor activation of the apoptotic pathway is important. Recently, evidence implicating necroptosis in neonatal brain injury was obtained; the RIP1 inhibitor necrostatin-1 reduces the formation of the RIP1-RIP3 complex and attenuates HI brain injury in postnatal day 7 male mice [27]. Necrostatin-1 also decreased the accumulation of oxidants, prevented the decline in complex I activity and improved ATP levels 24 h and 96 h after HI [44] supporting the hypothesis that execution of necroptosis in the immature brain depends on mitochondria.

## 5 Potential clinical translation

A variety of drugs targeting cell death pathways have been tested in animal models of perinatal brain injury. The amplitude of neuroprotection observed in these studies has been quite variable, and sometimes the results are inconsistent between models and research groups. However, several compounds (erythropoietin, N-acetyl-cysteine, caspase-2 inhibitors, p53 inhibitors, melatonin, JNK inhibitors) have shown promising neuroprotective properties [18], [22], [45], [46], [47], [48], [49].

The clinical translation is, however, hampered by several obstacles that have to be overcome. Firstly, it is not known whether all of the candidate drugs can cross the blood–brain barrier, but recent studies using dendrimers hold promise as a means of facilitating transfer across both blood brain barrier and across cell membranes [50]. Secondly, the immature brain undergoes major developmental changes that will determine the long-term cognitive and motor outcome, meaning that the safety of every compound needs to be carefully tested in long-term follow-up studies. Thirdly, most drugs tested are non-specific and have multiple effects that go beyond the anti-anti-apoptotic/anti-necrotic effects. Fourth, most interventions have been performed in a limited number of rodent models and validation in gyrencephalic animals or in humans is lacking. Finally, hypothermia is now used in clinical practice as a neuroprotectant for term hypoxic–ischemic encephalopathy. This means that drugs need to be tested for their neuroprotective efficacy in combination with hypothermia [45] rather than given alone which is not done in most experimental studies.

There are two drugs, melatonin and erythropoietin, which have been tested in clinical trials in preterm infants, and in term infants in conjunction with hypothermia. A recent randomized trial based on a relatively small number of patients has shown that preterm infants had a significantly better cognitive outcome after erythropoietin vs. placebo [51]. Furthermore, in a large randomized clinical trial, exposure of preterm infants to high dose of erythropoietin was associated with significantly reduced brain damage on MRI [52]. While awaiting the results of several ongoing promising trials, future research will aim at defining more targeted approaches considering the critical role of mitochondria for apoptotic and necrotic cell death and how these pathways may be different in males and females [53].

## 6 Summary

Mitochondria are center stage in the response to HI in the neonatal brain. Mitochondrial impairment leads to bioenergetic failure, generation of reactive oxygen species and dysregulation of calcium homeostasis culminating in Bax-dependent mitochondrial permeabilization and apoptotic cell death. In addition, death receptors are activated that could lead to caspase-8 dependent cell death or triggering of necroptosis through the formation of a necrosome, composed of RIP1 and RIP3. Recruitment of MLKL targets the necrosome to associate with plasma and mitochondria/endoplasmic reticulum membranes and RIP3 induces mitochondrial fission, excessive reactive oxygen species production and cell death with a predominately necrotic phenotype.
